# Colon Capsule Endoscopy as a Diagnostic Adjunct in Patients with Symptoms from the Lower Gastrointestinal Tract

**DOI:** 10.3390/diagnostics11091671

**Published:** 2021-09-13

**Authors:** Thomas Bjørsum-Meyer, Gunnar Baatrup, Anastasios Koulaouzidis

**Affiliations:** 1 Department of Surgery, Odense University Hospital, 5700 Svendborg, Denmark; gunnar.baatrup@rsyd.dk; 2 Department of Clinical Research, University of Southern Denmark, 5230 Odense M, Denmark; 3 Department of Social Medicine & Public Health, Pomeranian Medical University, 70-204 Szczecin, Poland; akoulaouzidis@hotmail.com

Prompted by the core idea of wireless capsule endoscopy as a painless gastrointestinal examination, and the easy adoption of small bowel capsule endoscopy, the armamentarium of the capsule-based imaging platforms has grown to include colon capsule devices as well [1]. Fueled to an extent by technology can-do and manufacturers’ targets, this process seems to have ignored advice from clinicians as well as aspirations for precision medicine, i.e., the need-do and need-have of such devices. We have been handed a tool with certain specifications and the initial effort was consumed in an a priori lost fight, i.e., to prove that CCE has equivalent clinical validity to colonoscopy. Paradoxically, we have to thank a major epidemiologic crisis for exploring alternative uses of this tool, e.g., as part of a stepped triage pathway. In a recent meta-analysis by Pin-Vieito et al. on the performance of a fecal immunochemical test (FIT) in symptomatic patients presenting to primary health care, they concluded that the FIT is the test of choice for patients with new-onset complaints from the lower gastrointestinal (GI) tract [2]. The triage with the FIT has undoubtedly high potential although more data are warranted, especially about cut-off levels and consequences. However, we find a wide adoption of this strategy in general practice potentially hazardous and express our concern. More studies have reported poor detection rates of the FIT for Union for International Cancer Control (UICC) stage I colorectal cancer (CRC) and specifically T1 cancers in screening settings [3,4]. The same findings must be suspected in symptomatic patients. In a recent study in a screening population, Niedermaier et al. found that decreasing the FIT cut-off value from 40 µg/g to 10 µg/g increased the sensitivity from 37% to 61% [5]. On the other hand, such a change would increase the number of colonoscopies almost threefold. We need to find a delicate balance between not missing cancers without overburdening already tied-up endoscopy units. Pin-Vieito et al. raise a very important yet unsettled obstacle: how do we decrease the number of FIT negative interval cancers without deluging overburdened endoscopy units? In our opinion, we need a sustainable adjunct to the FIT in order not to cut corners and without exhausting hospital services. We need a diagnostic modality which can be performed in primary health care. Colon capsule endoscopy (CCE) might be part of such a solution/pathway. CCE got wind in its sails during the COVID-19 pandemic as it can be performed in out-patient clinics with minimal contact with health care professionals and other patients [6]. Several studies have reported CCE to have sensitivity and specificity for detecting CRC and significant polyps (cancer precursors) similar to or better than conventional colonoscopy (CC) [7,8].

In a recent UK study, the diagnostic performance of FIT was evaluated in a population with low-risk symptoms of CRC in primary care and found the FIT to perform very well in triage patients [9]. The authors reported a sensitivity and specificity for CRC of 84.3% and 85.0%, respectively, at a cut-off value of 10 µg Hb/g feces. They estimated that a FIT value of 37 µg Hb/g feces in an individual corresponds to a CRC risk of 3%. This equals the risk for which an urgent colonoscopy is warranted according to the NICE recommendations (https://www.nice.org.uk/guidance/NG12, accessed on 10 August 2021). Based on these estimates, we have proposed a flowchart for handling patients presenting at their general practitioner (GP) with symptoms from the lower GI based on the result of the FIT (Figure 1).

CCE has shortcomings that need to be mentioned to understand its current position as a diagnostic tool. Before CCE, extensive bowel preparation is required to achieve an adequate visualization of the bowel mucosa as gas insufflation and washing/suction are not options, compared to CC. Further improvements in bowel cleansing regimens for CCE are requisite to make it an equal diagnostic alternative to CC. Apart from requiring considerable health care resources, CC is also unpleasant for patients with risks of severe complications in terms of bowel perforation and post-polypectomy bleeding. Not least, the procedure often induces embarrassment and discomfort in the patients. In our opinion, we are in urgent need of a patient-friendly, out-of-hospital diagnostic modality which can be initiated and concluded by the GP to alleviate overburdened endoscopic units without increasing the rates of missed significant pathology. Due to recent advances in artificial intelligence (AI)-assisted CCE, the tool we need seems just around the corner. AI-based systems have been developed and found to automatically detect colorectal neoplasia with high accuracy [10]. AI-based algorithms for size estimation and characterization of colorectal neoplasia are under development. They are expected to be implemented very soon in an automatized analysis of CCE videos, providing a result within minutes after egestion of the colon capsule. This would enable the GP to reassure the patients or refer to CC for polyp removal or urgent biopsy in due time. As most symptomatic patients leave endoscopic units after a colonoscopy without achieving a clear cause of their symptoms, there is an obvious need for improvement to allocate patients with lower GI symptoms to the proper examination without unnecessary delay. Despite a wider adoption of CCE hastened by the COVID-19 pandemic, CC remains the preferred diagnostic modality in screening settings, although the CCE performance in bowel cancer screening is currently under evaluation in an ongoing clinical trial [11].

## Figures and Tables

**Figure 1:**
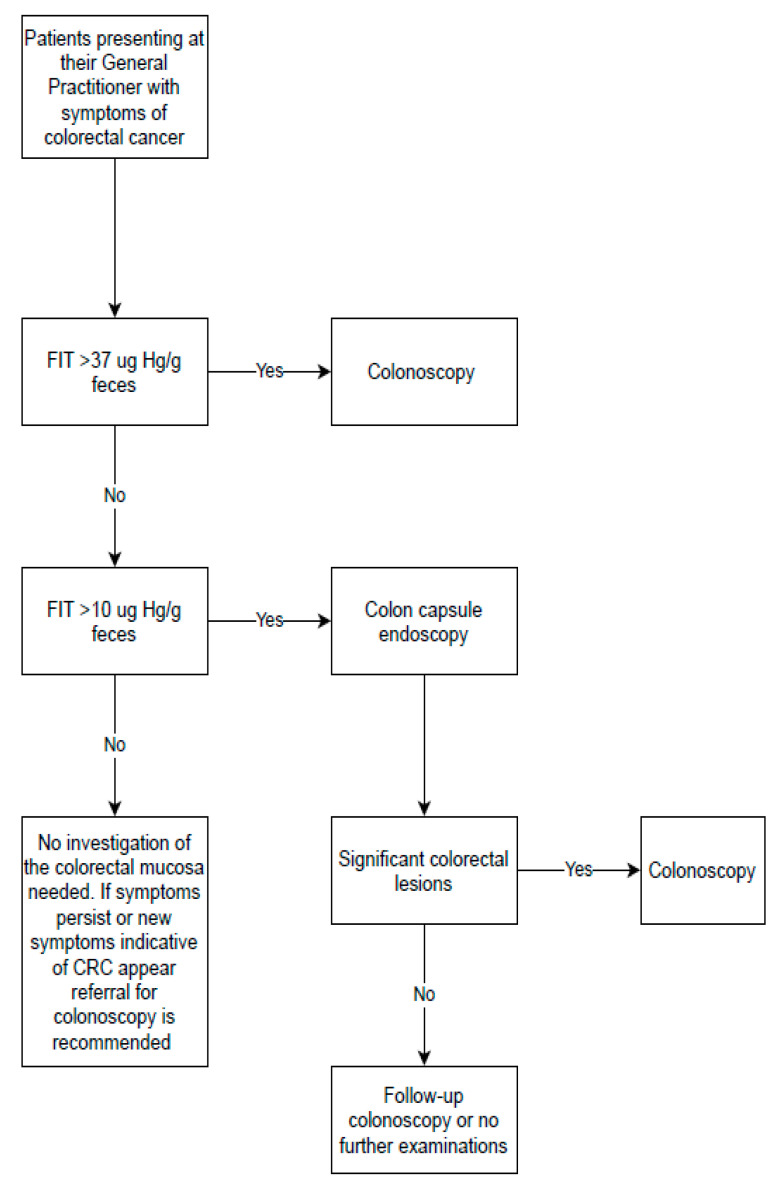
Suggested flow of patients presenting with symptoms of colorectal cancer at their general practitioner. Definitions: significant colorectal lesions: suspected cancer or any polyps > 9 mm.

## Data Availability

Not applicable.
